# Drivers of infection with *Toxoplasma gondii* genotype type II in Eurasian red squirrels (*Sciurus vulgaris*)

**DOI:** 10.1186/s13071-023-06068-6

**Published:** 2024-01-23

**Authors:** Sara R. Wijburg, Margriet G. E. Montizaan, Marja J. L. Kik, Maike Joeres, Garance Cardron, Christine Luttermann, Miriam Maas, Pavlo Maksimov, Marieke Opsteegh, Gereon Schares

**Affiliations:** 1 Centre for Infectious Disease Control, Centre for Zoonoses and Environmental Microbiology, National Institute for Public Health and the Environment (RIVM), Antonie Van Leeuwenhoeklaan 9, 1, 3720 BA Bilthoven, The Netherlands; 2 Dutch Wildlife Health Centre, Faculty of Veterinary Medicine, University of Utrecht, Yalelaan 1, 3584 CL Utrecht, The Netherlands; 3 Department Biomolecular Health Sciences, Pathology, Veterinair Pathologisch Diagnostisch Centrum, Yalelaan 1, 3584 CL Utrecht, The Netherlands; 4 Institute of Epidemiology, Friedrich-Loeffler-Institut, Federal Research Institute for Animal Health, Südufer 10, 17493 Greifswald - Insel Riems, Germany; 5 Institute of Immunology, Friedrich-Loeffler-Institut, Federal Research Institute for Animal Health, Südufer 10, 17493 Greifswald - Insel Riems, Germany

**Keywords:** Toxoplasmosis, Zoonoses, Parasite, Oocyst, Sentinel, Monitoring, Squirrel, Microsatellite typing, Population structure

## Abstract

**Background:**

In September 2014, there was sudden upsurge in the number of Eurasian red squirrels (*Sciurus vulgaris*) found dead in the Netherlands. High infection levels with the parasite *Toxoplasma gondii* were demonstrated, but it was unclear what had caused this increase in cases of fatal toxoplasmosis. In the present study, we aimed to gain more knowledge on the pathology and prevalence of *T. gondii* infections in Eurasian red squirrels in the Netherlands, on the *T. gondii* genotypes present, and on the determinants of the spatiotemporal variability in these *T. gondii* infections. The presence of the closely related parasite *Hammondia hammondi* was also determined.

**Methods:**

Eurasian red squirrels that were found dead in the wild or that had died in wildlife rescue centres in the Netherlands over a period of seven years (2014–2020) were examined. Quantitative real-time polymerase chain reaction was conducted to analyse tissue samples for the presence of *T. gondii* and *H. hammondi* DNA. *Toxoplasma gondii*-positive samples were subjected to microsatellite typing and cluster analysis. A mixed logistic regression was used to identify climatic and other environmental predictors of *T. gondii* infection in the squirrels.

**Results:**

A total of 178 squirrels were examined (49/178 *T. gondii* positive, 5/178 *H. hammondi* positive). Inflammation of multiple organs was the cause of death in 29 squirrels, of which 24 were also *T. gondii* polymerase chain reaction positive. *Toxoplasma gondii* infection was positively associated with pneumonia and hepatitis. Microsatellite typing revealed only *T. gondii* type II alleles. *Toxoplasma gondii* infection rates showed a positive correlation with the number of days of heavy rainfall in the previous 12 months. Conversely, they showed a negative association with the number of hot days within the 2-week period preceding the sampling date, as well as with the percentage of deciduous forest cover at the sampling site.

**Conclusions:**

*Toxoplasma gondii* infection in the squirrels appeared to pose a significant risk of acute mortality. The *T. gondii* genotype detected in this study is commonly found across Europe. The reasons for the unusually high infection rates and severe symptoms of these squirrels from the Netherlands remain unclear. The prevalence of *T. gondii* in the deceased squirrels was linked to specific environmental factors. However, whether the increase in the number of dead squirrels indicated a higher environmental contamination with *T. gondii* oocysts has yet to be established.

**Graphical Abstract:**

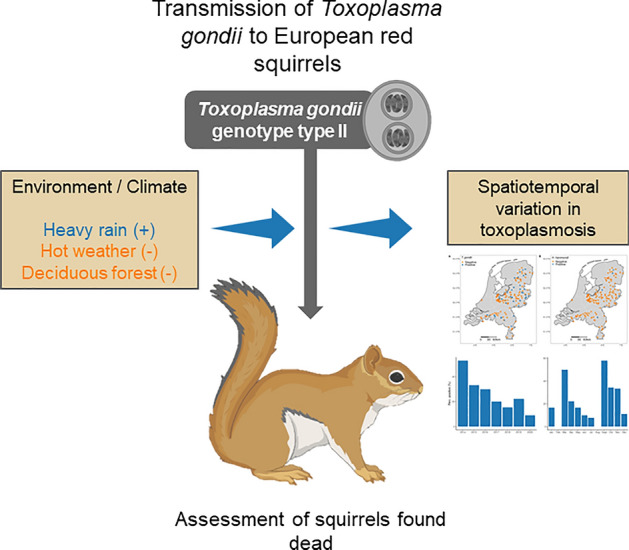

**Supplementary Information:**

The online version contains supplementary material, which is available at 10.1186/s13071-023-06068-6.

## Background

The protozoan parasite *Toxoplasma gondii* is the causative agent of toxoplasmosis, a widespread zoonotic disease that affects warm-blooded animals globally [1]. Felines, including domestic cats (*Felis catus*), European wildcats (*Felis silvestris*), and Eurasian lynxes (*Lynx lynx*), are its definitive hosts in Europe [2–5]. Following initial infection with *T. gondii*, felines excrete millions of environmentally resistant oocysts in their faeces [6, 7], which put other animals and humans at risk of exposure [8, 9]. Various species have been identified as intermediate hosts, with tissue cysts developing especially in their muscle tissues and brain [10]. *Toxoplasma gondii* infection in livestock can lead to substantial economic losses due to reproductive failure, e.g. in goats and sheep [11–13]. Animals intended for human consumption that harbour tissue cysts of *T. gondii* pose a risk to public health [14]. Humans can become infected through eating undercooked meat containing tissue cysts, by ingesting oocysts present in water, soil or on raw unwashed fruits and vegetables, and by congenital transmission [7, 15, 16]. Although most humans infected with *T. gondii* do not have specific symptoms, infection can cause severe disease, or even death in foetuses and immunocompromised individuals [17].

*Toxoplasma gondii* is also of concern for wildlife, as in certain species infection may be lethal [7, 18–25]. In September 2014, there was a sudden increase in the number of dead Eurasian red squirrels (*Sciurus vulgaris*) reported to the Dutch Wildlife Health Centre (DWHC) and the Dutch Mammal Society [26]. In 20 of the 37 squirrels examined, the cause of death (COD) was disseminated *T. gondii* infection [22]. There is limited information on *T. gondii* in squirrels [18, 23, 27], and it is unclear whether the cases of toxoplasmosis and the increase in the reported number of dead squirrels were related to climatic or other environmental factors, or whether specific genotypes of *T. gondii* with a higher virulence in squirrels may have been associated with the increase in the number of deaths [28]. Gaining knowledge about *T. gondii* infections in Eurasian red squirrels is not only important for the conservation of this species, which already suffers from various threats [29], but also from a One Health perspective, as the rate of toxoplasmosis in squirrels may represent a proxy for the exposure of humans living in the same areas as these animals to *T. gondii*.

To increase our understanding of *T. gondii* in red squirrels, we retrospectively examined data collected by the DWHC from 2014 to 2020. One objective of this study was to gain insight into the determinants of the spatiotemporal characteristics of the *T. gondii* infection rates in these squirrels. Another aim was to assess the pathology of *T. gondii* infection. To assess associations with pathogenicity, we compared the microsatellite (MS) genotypes of *T. gondii* in squirrels from the Netherlands with other MS genotypes observed in Europe. The presence of the parasite *Hammondia hammondi* was also determined, as it is a closely related parasite and has similar biology to *T. gondii*, which may lead to misdiagnosis of the infectious agent.

## Methods

### Study area and populations

Eurasian red squirrels that were found dead in the wild or that had died in wildlife rescue centres were collected by the DWHC between 2014 and 2020 to examine the COD (Additional file 4). A carcass was accepted for examination depending on its freshness (< 24 h since death), the time that the squirrel had spent in captivity in the rescue centre (< 24 h), and the capacity of the DWHC to examine the carcass. The number of squirrels examined by the DWHC was temporarily increased in 2019 and 2020 because squirrels were then accepted that had been dead for < 48 h or had been in captivity in rescue centres for < 48 h before death, and because the DWHC gave priority to squirrels over other animals at that time. The squirrels were sexed and weighed; age could not reliably be assessed. Geographical coordinates were determined based on the site of collection (i.e. coordinates were not based on the location of the rescue centre).

### Pathological and histopathological examination

Post-mortem examination included gross examination and cytological and histopathological analyses. Immunohistochemical, bacteriological and viral investigations were performed when feasible. Cytological examination was performed on the liver, spleen, lungs, and intestinal contents, which were stained with Hemacolor quick stain (Merck, Darmstadt, Germany). For histopathological examination, tissue samples of internal organs (brain, spleen, lung, liver, kidney, and heart) were fixed in 4% phosphate-buffered formalin, embedded in paraffin, cut into 4-μm sections and stained with hematoxylin and eosin. Duplicate slides were examined in the immunohistochemical assay, which used polyclonal antibodies against *T. gondii* (avidin–biotin complex immunoperoxidase stain, goat anti rabbit/biotin; Dako E0432; Klinipath, the Netherlands).

### Real-time polymerase chain reaction analyses

Lung, liver and heart samples were examined for *T. gondii* DNA by quantitative real-time polymerase chain reaction (qPCR) targeting a 529-base pair repetitive element, TgREP-529 [30, 31]. A quantitative real-time qPCR for *H. hammondi* was also performed [32]. DNA was extracted using the NucleoMag Tissue kit (Macherey–Nagel, Düren, Germany) in accordance with the manufacturer’s instructions with a previously described adjustment [33] whereby the volume of lysis buffer and proteinase K was adapted to the weight of the individual tissue samples, which ranged from 25 to 500 mg. Sequences of primers and probes (Eurofins, Ebersberg, Germany), and the final concentrations used are provided in Additional file 1: Table S1. To monitor qPCR inhibition, a heterologous plasmid DNA resembling the gene that encodes enhanced green fluorescent protein (EGFP) [34] was added to the reaction mix, which included the primers EGFP1-F and EGFP2-R and the probe EGFP1 [35]. The volume of the final quantitative real-time qPCR reaction was 25 μl, and a commercial master mix was used (5× PerfeCTa qPCR ToughMix; VWR, Darmstadt, Germany). Amplification was done on a CFX96 instrument (Bio-Rad, Munich, Germany).

### MS typing

For each *T. gondii*-positive animal [quantification cycle (Cq) values < 34], the sample with the lowest Cq value was assessed by MS typing [36]. Extracted DNA was amplified in a multiplex PCR [37], using 15 unlinked MS markers. These markers included eight typing markers (TUB2, W35, TgM-A, B18, B17, M33, IV.1, XI.1) and seven fingerprinting markers (M48, M102, N83, N82, AA, N61, N60). These fingerprinting markers display a high level of polymorphism within the clonal lineages type I, type II and type III [37]. Primers were used at a concentration 0.2 pmol/µl. The only divergence from the original method was that, in the case of M102, AA and N60, the fluorophore Atto 550 was used instead of NED to label amplicons during multiplex qPCR. MS typing followed recently described guidelines [36].

### Population genetic analyses of MS typing data

MS data conversion and processing were done using the R package adegenet (version 2.1.9) [38]. The genetic distance matrix for relative distances between MS genotypes was calculated using Bruvo’s method [39, 40]. In the computation of Bruvo’s distance, raw allele calls were divided by their respective repeat lengths, followed by rounding up. This methodology introduces a potential error when repeat lengths are of even magnitude, owing to adherence to standard IEC 60559 [41, 42]. The MS data were therefore normalized by employing R package poppr (version 2.9.3) [39]. This step was executed before the calculation of Bruvo’s distance to improve the accuracy of the results. Prior to the determination of clustering among the MS data, their clustering tendency was assessed by applying the Hopkins statistic [43, 44]. A Hopkins statistic value above 0.5 was used to conclude if the dataset had a significant clustering tendency [44]. Affinity propagation clustering (APC) was used to identify subpopulations from the *T. gondii* DNA samples for which MS data for all 15 markers were available. APC is an unsupervised classification model that clusters MS multi-locus genotypes (MLGs) according to their similarity, which is inferred by Bruvo’s distance [45]. To this end, Bruvo’s distance matrix was squared and converted by inverting the values [45]. The APC algorithm determines a single MLG from the set of input MLGs for each potential cluster that is most representative of that cluster. The optimal number of clusters was defined as the largest range of input parameters for which a constant number of clusters was calculated [45].

### Selection of spatiotemporal covariables

An overview of the explanatory variables is provided in Additional file 1: Table S2. To estimate the presence of domestic cats, four variables were chosen as indirect proxies (due to the limited population of wild cats solely present in the south of the province of Limburg, these species were not included [46]): annual estimate of the domestic cat population (determined by a public survey [47]; outcomes of that survey were available by personal communication with B. Beekhof, Nederlandse Voedingsindustrie Gezelschapsdieren) in the Netherlands per region [i.e. northern region (provinces of Drenthe, Friesland, and Groningen), eastern region (provinces of Gelderland and Overijssel), southern region (provinces of Limburg and North Brabant), western region (provinces of Flevoland, North Holland, Utrecht and South Holland) [48]); distance to the nearest urban area; human population density; and farm density (i.e. agriculture, horticulture, and livestock). The proportion of cats per region was assumed to remain stable over time. Distance to urban areas [functional land use map (BBG) category 2 [49]] was calculated as the Euclidean distance from the geolocation of the collected squirrel to the nearest urban area (metres). Both human population [50] and farm density [51] were calculated within a 3-km-diameter buffer zone (i.e. based on our estimation of cat and squirrel home ranges) centred around the geographical location where individual squirrels had been collected (Additional file 1: Table S2).

To explain the potential spatial variability of *T. gondii* infections, we considered landscape composition [8, 52], determined from data in the national land use database of the Netherlands (5 × 5-m^2^ resolution; Wageningen Environmental Research). Land cover was aggregated into seven categories: agricultural, urban, deciduous forest, coniferous forest, infrastructure, surface water, and nature reserve. The proportion per land cover type was calculated within a 3-km-diameter buffer zone. As infection with *T. gondii* may occur as a result of exposure to infective oocysts present in contaminated water [53], the nearest Euclidean distance (metres) from the geographical location of the collected squirrel to water bodies (BBG category 7 [49]) was also included (Additional file 2: Table S2).

Environmental infection pressure is determined by factors that affect the sporulation and survival of oocysts in the environment. To estimate the climatic parameters favourable for oocyst survival and sporulation, we retrieved interpolated data at 1-km^2^ spatial resolution from 34 weather stations in the Netherlands (Royal Netherlands Meteorological Institute) for 2013–2020 (Additional file 2: Table S2). These gridded layers were used to derive values [54] for the maximum length of a dry spell; the number of summer days when the maximum temperature was > 25 ℃ (SU25); the number of days with heavy rainfall (i.e. ≥ 25 mm) (R25mm); and the number of days when it froze (minimum temperature ≤ −6 ℃) (FD6) preceding the date of sampling of individual squirrels. The first three parameters were chosen based on the following hypotheses: oocyst survival is adversely affected by high temperatures and drought [55–57]; the survival of oocysts increases with levels of environmental moisture [58, 59]. Both sporulation and survival of oocysts are affected by low temperatures. Temperatures around 4 ℃, and most likely also temperatures below 4 ℃, have a negative effect on the survival of non-sporulated *T. gondii* oocysts; in one study [60], almost all non-sporulated oocysts died over a period of 2–3 months at temperatures around 4 ℃. Sporulated oocysts can survive at very low temperatures (down to −21 ℃) [2, 57, 61]. However, they are very susceptible to repeated freezing and thawing events [57], which are likely to occur under natural, non-experimental conditions. Moreover, sporulation of oocysts does not occur below ≤ −6 ℃, independent of moisture levels [62]. The climatic variables were calculated for the 5 × 5-km^2^ area around each individual squirrel geolocation point for the 2-week, 1-, 3-, 6-, and 12-month periods preceding the date of specimen collection (Additional file 1: Table S2).

Squirrels use food sources such as nuts, seeds, fruits, fungi, grains, vegetables, and roots, which requires a varying amount of time and activity on the ground. The ratios of the components of the diet of squirrels vary according to their availability [63]. We hypothesized that squirrels may have increased exposure to *T. gondii* in years with a higher Living Planet Index (LPI) of fungi compared to the general trend. This effect may be amplified if mast production is low in the same years. Mast year was determined for beech (*Fagus sylvatica*) and oak (*Quercus robur*) [64]. Data from the National Databank of Flora and Fauna were used to aggregate trends (LPI) for saprotrophic and ectomycorrhizal fungal species (*n* = 88), as described by van Strien et al. [65]. In short, the first step involved calculating the year-to-year degree of change per fungal species by dividing the index value for a given year by the index value of the preceding year [65]. Extreme increase was capped at 10, extreme decline was restricted to 0.1, and all instances where the value was < 1 were substituted by 1 [66]. Aggregated annual indices per region were calculated by using the yearly geometric mean of the degree of change. These geometric means were transformed to annual indices (LPI), with the first year (1994) set to 100 [65]. Trend lines were derived by using smoothed conditional means (Additional file 2: Figure S1).

### Statistical analysis

An exact binomial test was used to determine confidence intervals (CIs) for infection rates. The association between squirrel *T. gondii* infection status and sex were assessed using a chi-squared test. Univariable logistic regression was used to assess patterns over the years, between regions, and between seasons. A generalized linear mixed model (GLMM) analysis was performed to investigate associations between positivity for *T. gondii* and potential risk factors (Additional file 1: Table S2). A random year effect was used to account for any unobserved factors that may have differed between years and may have affected infection risk [67]. Pre-processing of data included standardization by using the* z*-score (e.g. the mean was subtracted from each observation and the result divided by the SD), exclusion of variables with zero variance, and the removal of observations for which values were missing [68]. We first built univariable mixed logistic regression models to select potential candidate variables for the final GLMM. Predictor variables with *P* ≤ 0.25 were retained as input variables for the final GLMM. Correlations between potential predictor variables were assessed [69]. Where variables were highly correlated (*r*_*s*_ > 0.6) or there was multicollinearity between predictor variables (variance inflation factor < 10) [70], the predictor variable with the lowest *P*-value in the univariable mixed logistic regression was retained (Additional file 2: Figure S2). Forward and backward model selection based on the corrected Akaike information criterion was conducted [71]. To measure the strength of associations, the odds ratio and the 95% CIs were determined. *P*-values below 0.05 were considered to indicate statistical significance.

### Software

Quantitative real-time qPCR results were analysed using CFX Manager software version 1.6 (Bio-Rad). MS data analyses, covariate data extraction, and statistical analyses were performed in R version 4.3.1.

## Results

**Fig. 1:**
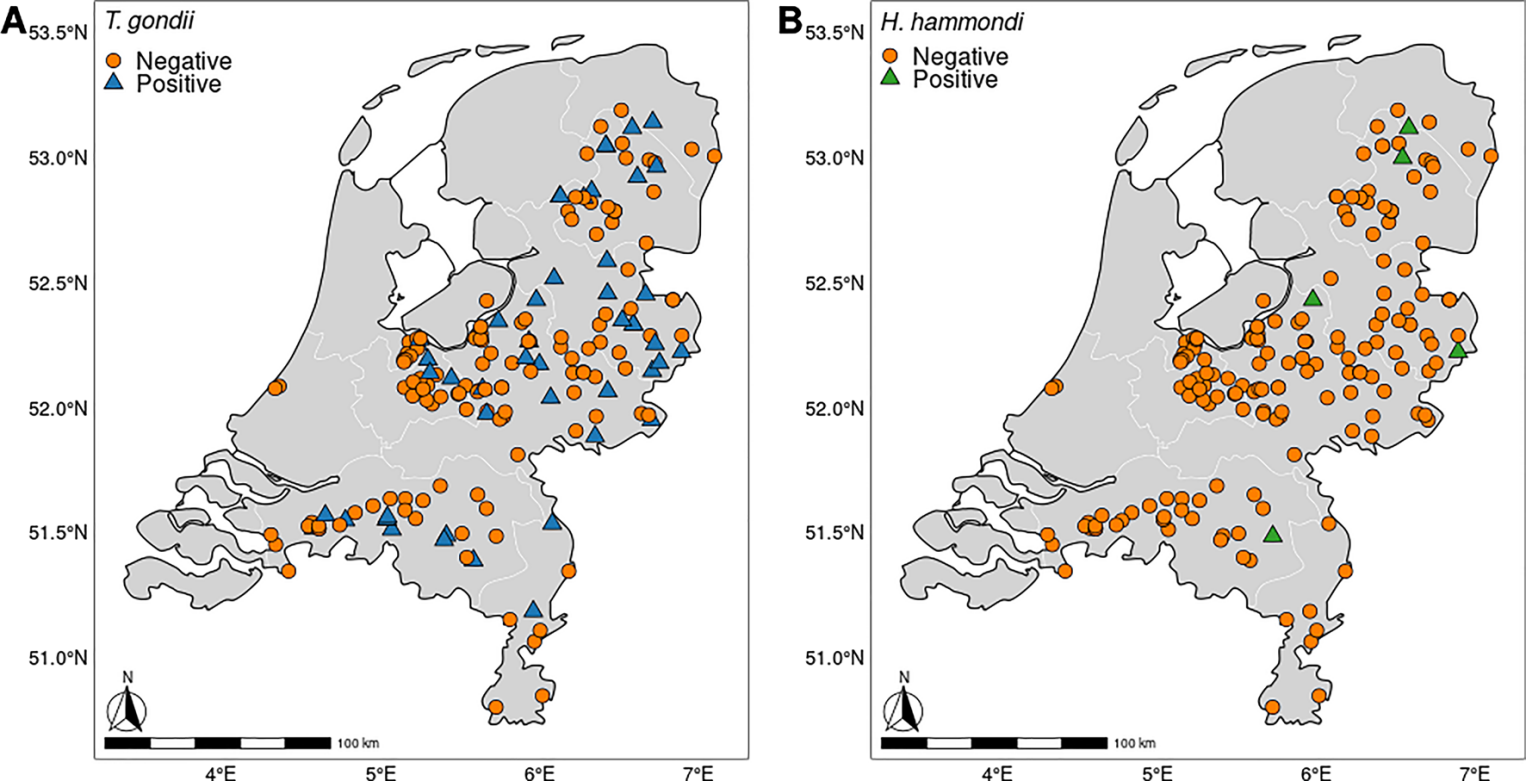
Geographical distribution of the squirrels that were quantitative real-time polymerase chain reaction (qPCR) positive or negative for *Toxoplasma gondii* (**A**) or *Hammondia hammondi* (**B**)

In total, 178 Eurasian red squirrels were tested for the presence of *T. gondii* and *H. hammondi* by quantitative real-time qPCR (Fig. 1). Of these squirrels, 27.5% (49/178, 95% CI 21.1–34.7%) tested positive for *T. gondii* and 2.8% (5/178, 95% CI 0.9–6.4%) for *H. hammondi* (Fig. 1B). Three of the squirrels were positive for both *H. hammondi* and *T. gondii*. Of the 174 specimens for which sex could be determined, 78 were female and 96 were male. The proportion of squirrels infected with *T. gondii* did not differ between the sexes (chi-squared test, χ^2^ = 0.022,* df* = 1, *P* = 0.88), and was 28.2% for females (95% CI 18.6–39.5%), and 26.0% for males (95% CI 17.6–36.0%).

*Toxoplasma gondii* infection rates significantly decreased over the years, with positivity ranging from 52.9% in 2014 to 9.1% in 2020. Seasonal differences were detected. In autumn, significantly more *T. gondii*-positive squirrels were observed in comparison to winter and summer. Autumn also represented the period in which the largest proportion [40.4% (72/178), 95% CI 33.2–48.0%] of squirrels had been collected. There was no evidence of regional differences in *T. gondii*-positive samples (Table 1; Additional file 1: Table S3). Since only five squirrels were positive for *H. hammondi*, and each of them had been collected in a different year and month (Additional file 1: Table S4), no statistical analyses were performed on their data.

**Table 1:** Overview of quantitative real-time polymerase chain reaction (qPCR) *Toxoplasma gondii*-positive squirrels (*n* = 178) per sampling year, season, and region, with odds ratios (*OR*) from univariable logistic regression analysis

	% Positive (*n* positive/*n*; 95% CI)	OR (95% CI)	*P*-value
Sampling year			
2014	52.9 (18/34; 35.3–70.2)	1	–
2015	33.3 (9/27; 16.5–54.0)	0.44 (0.15–1.24)	0.128
2016	30.0 (6/20; 11.9–54.3)	0.38 (0.11–1.19)	0.106
2017	20.0 (3/15; 4.3–48.1)	0.22 (0.04–0.85)	0.040*
2018	15.4 (2/13; 1.9–45.4)	0.16 (0.02–0.72)	0.030*
2019	22.2 (8/36; 10.1–39.1)	0.25 (0.09–0.70)	0.009*
2020	9.1 (3/33; 1.9–24.3)	0.09 (0.02–0.31)	< 0.001*
Season			
Autumn (September–November)	44.4 (32/72; 32.7–56.6)	1	–
Winter (December–February)	8.0 (2/25; 0.98–26.0)	0.11 (0.02–0.40)	0.004*
Spring (March–May)	27.1 (13/48; 15.3–41.8)	0.46 (0.21–1.01)	0.056
Summer (June–August)	6.1 (2/33; 0.74–20.2)	0.08 (0.01–0.29)	0.001*
Region			
East	29.9 (23/77; 20.0–41.3)	1	–
South	28.2 (11/39; 15.0–44.9)	0.92 (0.38–2.13)	0.852
West	16.7 (5/30; 5.6–34.7)	0.47 (0.14–1.30)	0.169
North	31.2 (10/32; 16.1–50.0)	1.07 (0.42–2.57)	0.886

*CI* Confidence interval

* *P* < 0.05

### Pathological and histopathological examination

**Fig. 2:**
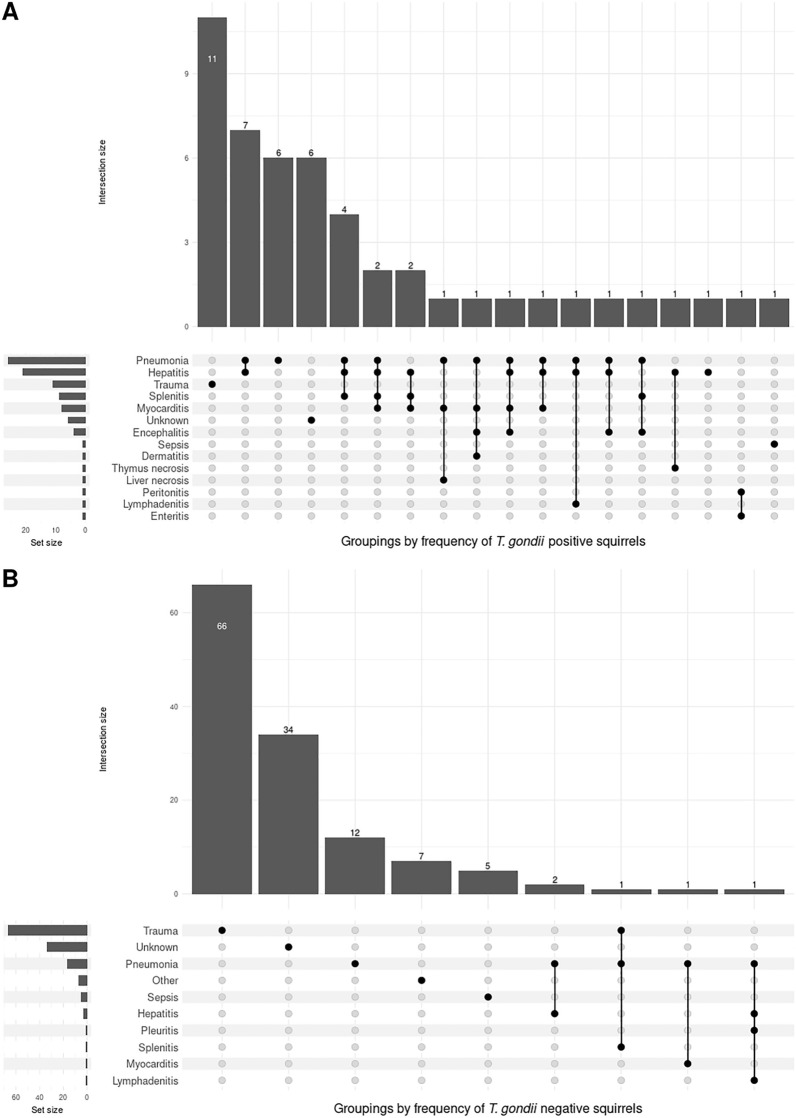
Cause of death determined from pathological examination of quantitative real-time polymerase chain reaction (qPCR) *Toxoplasma gondii*-positive squirrels (**A**) and real-time qPCR *T. gondii*-negative squirrels (**B**). Connections between dots indicate co-occurring conditions

In 24 of the 49 squirrels (49.0%) that tested positive for *T. gondii* by quantitative real-time qPCR, inflammation of multiple organs (i.e. inflammation of two or more organs) was determined to be the main COD (Fig. 2A). A combination of the following conditions was detected: hepatitis (20/24), pneumonia (20/24), splenitis (9/24), myocarditis (8/24), encephalitis (4/24), peritonitis (1/24), lymphadenitis (1/24), dermatitis (1/24), liver necrosis (1/24), enteritis (1/24), and thymus necrosis (1/24). Pneumonia-only was detected in 12.2% (6/49) of the positive animals, hepatitis-only in 2.0% (1/49), and sepsis in 2.0% (1/49). For six animals the COD was unknown. *Toxoplasma gondii* infection was positively associated with pneumonia (chi-squared test, χ^2^ = 46.6,* df* = 1, *P* < 0.001), and with hepatitis (chi-squared test, χ^2^ = 28.7,* df* = 1, *P* < 0.001). In all 24 squirrels with inflammation of two or more organs, the inflammation was acute, i.e. there was no visible signs of fibrosis. Trauma was the COD in 22.4% (11/49) of the 49 squirrels, and no inflammation was seen that could have been the COD.

In *T. gondii*-negative animals, trauma was shown to be the leading COD (51.2%, 66/129; Fig. 2B). In the remaining animals, the COD included pneumonia (9.3%, 12/129), inflammation of multiple organs (3.9%, 5/129), and sepsis (3.9%, 5/129). In five squirrels with inflammation of multiple organs combinations of the following conditions were observed: hepatitis (3/5), lymphadenitis (1/5), myocarditis (1/5), pleuritis (1/5), pneumonia (5/5), and splenitis (1/5). In 34 animals the COD could not be determined (26.4%, 34/129). Further details on the COD are provided in Additional file 1: Table S5.

In the *T. gondii*-positive animals, the trachea often contained foam, and the lungs were hyperemic and oedematous. In general, the liver was enlarged and pale, and the spleen was also enlarged. Pulmonary interstitial lymphoplasmacytic and neutrophilic infiltrates with oedema and numerous intra-alveolar macrophages were also revealed. The *T. gondii*-negative animals showed different types of infiltrates, which were not consistently noted. 

Data from immunohistochemical analyses (IHC) were evaluated for a total of 56 squirrels. In 43 of these, IHC indicated a *T. gondii* infection. In 15 of these 43 squirrels (34.9%), *T. gondii* infection was confirmed by quantitative real-time qPCR (Additional file 1: Table S5). One squirrel shown to be positive for *T. gondii* by quantitative real-time qPCR was not shown to be infected according to the IHC results. One squirrel considered to be positive for *T. gondii* according to the IHC results was shown to be negative for the parasite by quantitative real-time qPCR but positive for *H. hammondi* (Additional file 1: Tables S6, S7). Four of the five quantitative real-time qPCR-positive *H. hammondi* samples were not subjected to IHC testing. The remaining sample was shown to be positive for *T. gondii* by IHC.

### *Toxoplasma gondii* and *H. hammondi* detection per organ

Thirty-five of the 49 *T. gondii*-positive squirrels tested positive for all organs. In these 35 squirrels, the Cq values for the heart, lung and liver correlated to each other (Additional file 2: Figure S6). In 33 of these squirrels, the Cq-values ranged between 15 and 25, whereas in two of the squirrels the Cq value was around 30 or above.

**Fig. 3:**
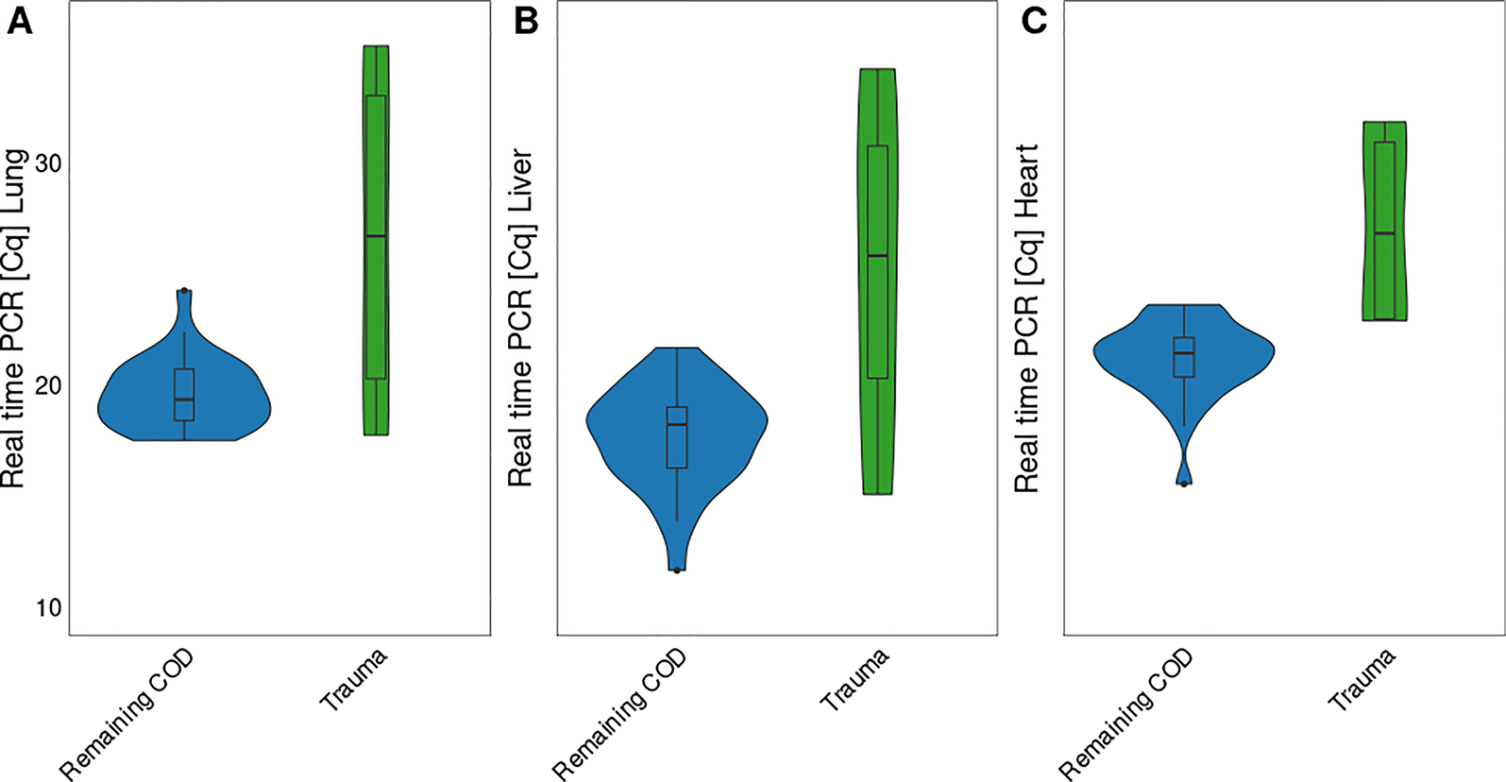
Quantification cycle (*Cq*) values of *Toxoplasma gondii* by quantitative real-time polymerase chain reaction (qPCR) for cause of death (*COD*) [comparison of *Trauma* and other (*Remaining*) CODs; these included inflammation of multiple organs, pneumonia, and undetermined causes] for individual organs [lung (*n* = 35) (**A**); liver (*n* = 35) (**B**); heart (*n* = 35) (**C**)]

Cq values were higher for squirrels that died from trauma compared to the other CODs (Wilcoxon rank sum test,* Z* = −3.51, *P* < 0.001; Fig. 3). Cq values were lower for the liver [median, 18.3 (interquartile range (IQR), 3.3)] than for the lung [median, 19.4 (IQR, 2.5); Wilcoxon rank sum test,* Z* = −3.42, *P* < 0.001]. Cq values for heart samples [median, 21.6 (IQR 2.2)] were higher than those for the liver (Wilcoxon rank sum test,* Z* = −4.53, *P* < 0.001) and lung samples (Wilcoxon rank sum test,* Z* = −3.66, *P* < 0.001; Additional file 2: Figure S3).

Of the remaining 14 squirrels, seven tested positive for *T. gondii* in the lungs only (14.3%, 7/49); one squirrel in the liver only (2.0%, 1/49); and one squirrel in the heart only (2.0%, 1/49). Four squirrels tested positive in the lungs and liver (8.2%, 4/49), but not in the heart (for one squirrel no heart sample was available). One squirrel tested positive in both heart and liver samples; no lung sample was available for this individual (Additional file 2: Figures S4, S5).

In four of the five squirrels that tested positive for *H. hammondi*, only the heart samples were positive (Additional file 2: Figures S4, S5). The remaining squirrel tested positive in the lungs (20.0%, 1/5). The Cq value for *H. hammondi* infections ranged from 30.2 to 31.5 for the heart samples, and was 34.4 for the lung sample.

### Population genetic analyses of *T. gondii* MS typing data

MS typing was used to analyse samples of 44 of the 49 *T. gondii*-positive squirrels. Five samples with a Cq value above 35 were impossible to type. Thirty-nine samples could be typed completely, and were shown to be *T. gondii* type II (Table 2; Additional file 3). Five samples were partially typable and showed only *T. gondii* type II alleles (Table 2; Additional file 3). By MS fingerprinting a total of 32 different MS types were observed among the 39 *T. gondii* for which the DNA was completely typed (Additional file 3). The value of the Hopkins statistic determined for the squirrel MS data, 0.65, strongly supported rejection of the null hypothesis positing the absence of a clustering tendency within the dataset.

**Table 2:** Characteristics of the 15 microsatellite (MS) markers used in this study, displayed for completely (*n* = 39) or partially (*n* = 5) typable *Toxoplasma gondii* samples

	Marker	Alleles	
		Completely typable	Partially typable
Typing markers	B18	158	158
	M33	169	169
	TUB2	289	289
	XI.1	356	356
	TgM-A	207	207
	W35	242	242
	IV.1	272–274	274
	B17	336	336
Fingerprinting markers	N61	85–117	95–101
	M48	211–235	219–227
	N83	308–316	310–312
	N82	109–123	111
	N60	140—144	140—142
	M102	172—182	174–182
	AA	259–289	261–271

For an overview of the population genetic analyses of *Toxoplasma gondii* MS data, see Additional file 3

**Fig. 4:**
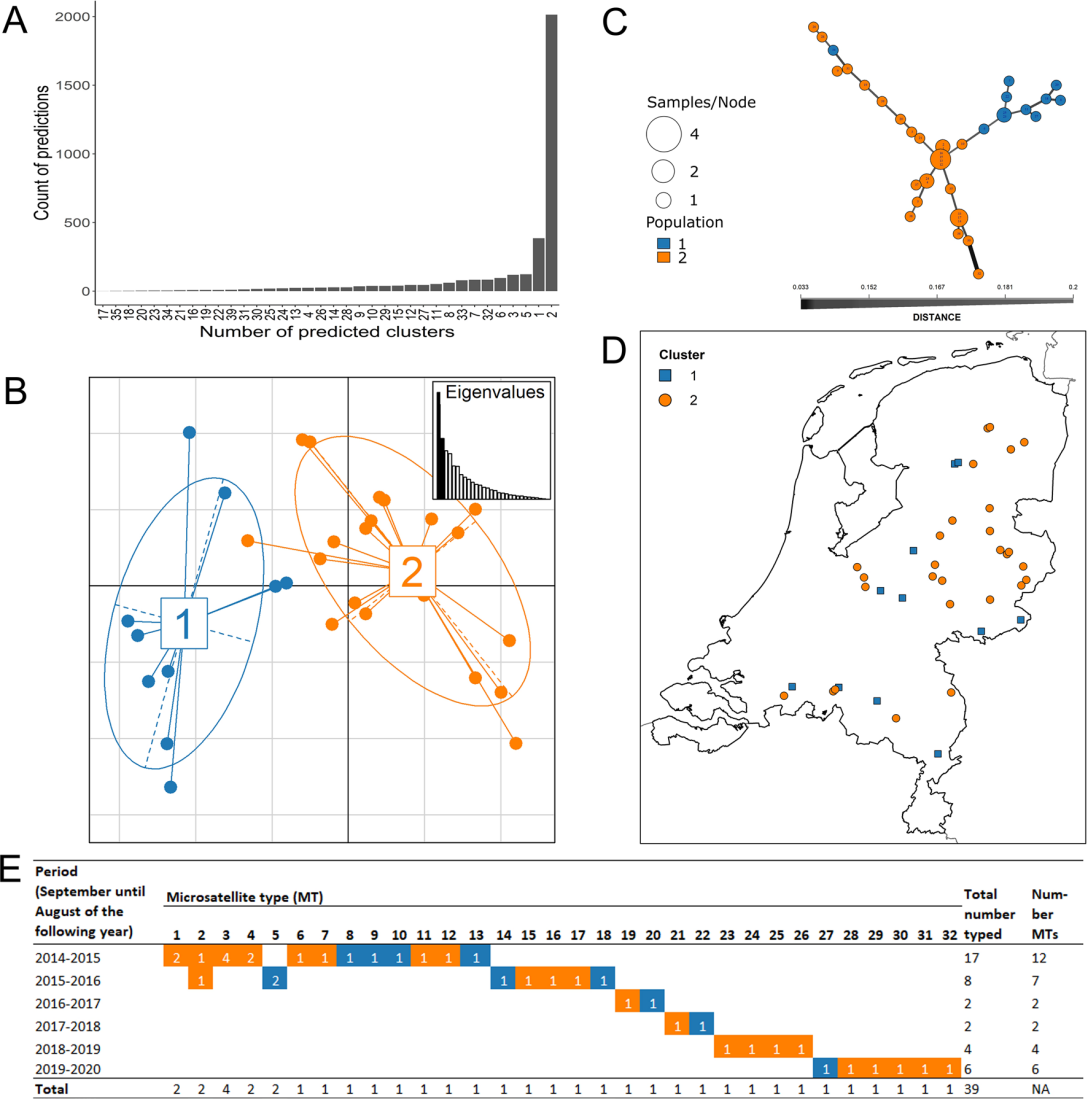
**A**–**E** Results of affinity propagation clustering (APC) using microsatellite (MS) genotyping data (*n* = 39), based on Bruvo's distance. **A** Variation in the number of parasite clusters indicated by APC. **B** Results of the principal coordinate analysis (colours correspond to the two clusters identified by APC). **C** Minimum spanning network of haplotypes (colour denotes parasite clusters according to APC; circle size corresponds to the total number of individuals with the same MS type; branch thickness is proportional to inferred genetic distance between haplotypes). **D** Geographic distribution of *Toxoplasma gondii*-positive squirrels (*n* = 39) harbouring the *T. gondii* MS types (*n* = 32; colours correspond to those of the clusters in **B**). **E** Representatives of the parasite clusters over time. Representatives of each of the clusters were observed for all of the periods, except for 2018–2019, for which there were only four typed samples

APC revealed two clusters of *T. gondii* type II in the sampled squirrels (Additional file 3; Fig. 4A). Principal coordinate analysis, based on Bruvo’s distances separated by the principal coordinates with the highest eigenvalues, i.e. principal coordinate 1 and principal coordinate 2, explaining 20.3% and 11.6% of the variation, respectively (Fig. 4B), and the minimum spanning network (MSN) of the haplotypes (Fig. 4C) revealed that the clusters were well separated from each other. The MSN was inferred by assuming the principle of parsimony, and was consistent with the clustering indicated by the APC. Representatives of both clusters were observed throughout the Netherlands (Fig. 4D). There was no statistical difference in geographical location of representatives of these two *T. gondii* clusters over time (Fig. 4E).

When the APC analysis included clonal *T. gondii* type II MS typing results for European type II genotypes that had been reported in a recent review (*n* = 444; Additional file 3) [28] in addition to those of the present study (*n* = 39), three clusters were determined (designated EurCluster1, EurCluster2, EurCluster3; Table 3; Additional file 3). The clustering tendency of the merged MS data was confirmed as statistically significant by the Hopkins statistic (0.9). A comparison of the proportions of these clusters among various northwestern European countries (Table 3) showed no significant differences between the following: France, Belgium, Netherlands, Germany, Denmark, and Norway (chi-square test, χ^2^ = 9.44,* df* = 12, *P* = 0.665).

**Table 3:** Proportions of three European clusters (EurCluster1, EurCluster2, EurCluster3) of clonal *Toxoplasma gondii* type II determined by affinity propagation clustering of merged MS data^a^

Country	EurCluster1	EurCluster2	EurCluster3
Portugal	9% (1/11)	0% (0/11)	*91*% (10/11)
Spain	0% (0/1)	*100*% (1/1)	0% (0/1)
England	0% (0/1)	*100*% (1/1)	0% (0/1)
France	26% (78/296)	*37*% (108/296)	*37*% (110/296)
Belgium	21% (6/28)	*43*% (12/28)	*36*% (10/28)
The Netherlands	22% (9/39)	*39*% (15/39)	*39*% (15/39)
Germany	15% (3/20)	*40*% (8/20)	*45*% (9/20)
Norway	0% (0/5)	*60*% (3/5)	*40*% (2/5)
Denmark	0% (0/12)	*50*% (6/12)	*50*% (6/12)
Austria	*63*% (38/60)	28% (17/60)	8% (5/60)
Czech Republic	*100*% (4/4)	0% (0/4)	0% (0/4)
Romania	0% (0/6)	17% (1/6)	*83*% (5/6)

^a^Proportions > 30% are shown  italic

### Predictor variables of *T. gondii* in the dead squirrels

The variables identified by univariable mixed logistic regression as putative predictor variables of infection with *T. gondii* in GLMM were the following: human population density, distance to nearest urban area, percentage of urban area, percentage of deciduous forest, distance to nearest water body, maximum length of dry spell (in the previous 2 weeks), SU25 (in the previous 2 weeks), R25mm (in the previous 12 months), and FD6 (in the previous 6 months) (Table 4; Additional file 1: Tables S2, S8). As percentage of infrastructure, SU25 (in the previous month), and FD6 (in the previous 12 months) were highly correlated to other predictor variables, they were excluded from further analyses (Table 4; Additional file 2: Figure S2). After model selection, the following predictors were determined to be significantly associated with *T. gondii* infection: percentage of deciduous forest at the sampling site (negative association), R25mm (in the previous 12 months; positive association), and SU25 (in the previous 2 weeks; negative association) (Table 4; Additional file 1: Table S9).

**Table 4:** Overview of univariable mixed logistic regression (*P* < 0.25) and final mixed logistic regression using data on squirrels (*n* = 175) from the Netherlands to identify predictors for* Toxoplasma gondii*-positive findings by quantitative real-time polymerase chain reaction (qPCR)

	Univariable regression		Final mixed logistic regression^d^	
Predictor variables^a^	*P*-value	OR (95% CI)	*P*-value	OR (95% CI)
Human population density	0.153	1.28 (0.91–1.79)	–	–
Nearest distance to urban areas	0.055	0.58 (0.34–1.01)	0.098	0.60 (0.33–1.10)
Land use-% Urban	0.151	1.29 (0.91–1.81)	–	–
Land use-% Deciduous forest	0.101	0.72 (0.49–1.07)	*0.020*	*0.60* (*0.39–0.92*)
Land use-% Infrastructure^b^	0.193	1.26 (0.89–1.77)	–	–
Distance to nearest water body	0.031*	0.60 (0.38–0.96)	–	–
Maximum length of dry spell–2 weeks^c^	0.170	1.27 (0.90–1.78)	0.113	1.36 (0.93–2.00)
R25mm–12 months	0.031*	1.48 (1.04–2.11)	*0.037*	*1.58* (*1.03–2.43*)
SU25–2 weeks	0.007*	0.31 (0.13–0.72)	*0.008*	*0.27* (*0.10–0.71*)
SU25–1 month^b^	0.020*	0.51 (0.29–0.90)	–	–
FD6–6 months	0.047*	0.56 (0.32–0.99)	0.144	0.62 (0.33–1.17)
FD6–12 months^b^	0.236	0.74 (0.45–1.22)	–	–
Mast year–Oak	0.073	0.65 (0.41–1.04)	0.302	0.75 (0.44–1.29)

*R25mm–12* *months* Days with heavy rainfall (≥ 25 mm) in the 12 months preceding sampling;* SU25–2 weeks* Summer days with maximum temperature > 25 ℃ in the 2 weeks preceding sampling;* SU25–1 month* summer days with maximum temperature > 25 ℃ in the month preceding sampling;* FD6–6 months* Days when it froze (minimum temperature ≤ −6 ℃) in the 6 months preceding sampling;* FD6–12 months* days when it froze (minimum temperature ≤ −6 ℃) in the 12 months preceding sampling

* *P* < 0.05

^a^For further details on predictor variables, see Additional file 1: Table S2

^b^*r*_*s*_ > 0.6; for further details, see Additional file 2: Figure S2

^c^Dry days in the 2-week period preceding the sampling date

^d^Model random effects, σ^2^ (random effect variance)—3.29; marginal *R*^2^/conditional *R*^2^—0.478/0.504

## Discussion

The primary objectives of this study were to enhance our understanding of the drivers of *T. gondii* infection in Eurasian red squirrels found dead in the Netherlands following the *T. gondii* outbreak of 2014, to assess the pathology of the infections, and to compare MS genotypes of *T. gondii* from the Netherlands with those observed in other European countries. We detected an overall *T. gondii* infection rate of 27.5%. Infection rates decreased over the years, but it remains to be determined whether this was due to declining environmental pressure or other, non-investigated, factors. There were no marked differences between regions, which possibly reflects the widespread geographic distribution of *T. gondii* in domestic cats in the Netherlands. 

The five animals that tested positive for *H. hammondi* represent, to the best of our knowledge, the first records of *H. hammondi* infection in squirrels [72]. In its other intermediate host species, *H. hammondi* is typically not virulent [73]. Two of these five squirrels, including the one in which the parasite was only detected in the lung, were thought to have died of trauma, while hepatitis and pneumonia were thought to have been the COD in two of the remaining animals and sepsis in the other one. Because *H. hammondi* DNA was only observed in the heart samples of four of these animals, and in the lung in the remaining one, it is very unlikely that *H. hammondi* infection contributed to their deaths.

Inflammation of multiple organs was the leading COD in *T. gondii*-positive animals (49.0%), followed by trauma (22.4%), and pneumonia (12.2%). In contrast, in *T. gondii*-negative animals, the leading COD was trauma (51.2%), followed by pneumonia (9.3%), and inflammation of multiple organs (3.9%). Pathological examination of the *T. gondii*-positive animals predominantly revealed pneumonia and hepatitis, which are signs of disseminated/acute toxoplasmosis [74]. Although lesions in the liver and lungs of red squirrels infected with *T. gondii* have been reported [24, 75, 76], in the present work conclusions must be drawn with care due to the design of the study. Only red squirrels that were found dead or that had died after being rescued and taken to wildlife rescue centres were examined. Thus, although *T. gondii* infection appears to have played a role in acute mortality in the squirrels, we cannot draw any firm conclusions on its lethality as we do not know how many squirrels survived infection with this parasite. Our results do, however, suggest that red squirrels are highly susceptible to infection with *T. gondii* type II. Therefore, it would be interesting to further investigate whether squirrel mortality due to *T. gondii* infection is indicative of a high environmental load of the parasite, as this could provide valuable insights into *T. gondii*-contaminated areas and consequently information of relevance to public health.

IHC appeared to be less specific than quantitative real-time qPCR for the detection of *T. gondii*. Only 15 of the 43 *T. gondii*-positive infections shown by IHC were confirmed by quantitative real-time qPCR. Additionally, one squirrel that was *T. gondii*-positive according to IHC but negative according to the results of quantitative real-time qPCR tested positive by qPCR for *H. hammondi*, which suggests that the parasite was misidentified by IHC [77]. As the morphology and antigenic composition of *T. gondii* and *H. hammondi* are similar [78], molecular tools are required for their differentiation [79]. The sensitivity of both IHC and quantitative real-time qPCR are lower when the *T. gondii* burden is low [80]. Both methods perform better in cases of disseminated/acute toxoplasmosis than in cases of chronic infection with unevenly distributed tissue cysts. Of the 49 squirrels testing positive for *T. gondii* by qPCR, 35 tested positive for three organs, five tested positive for two organs, and nine for one organ only. In *T. gondii*-negative individuals or organs, cysts may have been present at concentrations below the limit of detection [81]. Sampling multiple organs of an individual animal reduces the chance of false negatives. Due to the retrospective character of this study, it was not possible to use tissue cyst-specific antibodies to re-examine tissue sections for *T. gondii*.

The surprisingly high concentration of *T. gondii* DNA in most of the squirrels, which indicated a high infection load, undoubtedly contributed to the high success rate of the MS typing. The success rate was, to the best of our knowledge, exceptionally high for wildlife specimens [28]. The MS typing indicated the presence of *T. gondii* type II in the squirrels, which is the prevailing *T. gondii* lineage in both wild and domestic animals in Europe [82, 83]. Only *T. gondii* type II alleles were detected in studies undertaken in the Netherlands that examined samples from sheep [84] and squirrels [22], though the results were based on sequencing of the *GRA6* gene only. The two *T. gondii* type II clusters observed in the present study for squirrels from the Netherlands are in agreement with those identified for Europe (EurClusters 1-3) [28] and thus are not thought to represent specific genotypic entities.

Cases of *Toxoplasma gondii* were identified year-round, but both the number of reported dead squirrels and *T. gondii* infection rates peaked in autumn. A study undertaken in a neighbouring country [85] revealed that the proportion of domestic cats shedding *T. gondii* oocysts varied over the year. The reasons for this pattern were not entirely clear, but the seasonality of oocysts shedding could be modelled using climatic data [85]. Interestingly, the pattern of *T. gondii* oocysts shedding by felines in the earlier study [85] appeared to align with the increased rates of *T. gondii* infections in squirrels in autumn observed in this study. Moderate temperatures and high levels of humidity can promote the survival and sporulation of oocysts in the environment [86, 87]. In the present study, *T. gondii* infection was positively associated with heavy rainfall over the 12 months preceding sampling and negatively associated with hot summer days during the 2-week period preceding sampling. Our results also indicated that *T. gondii* infection was negatively associated with the percentage of deciduous forest in a 3-km-diameter buffer zone arround the site of sampling; however, this particular association is difficult to interpret. Deciduous forest cover was correlated with lower human density and possibly fewer cats; however, the effect of this association may be partly accounted for by inclusion of distance to urban areas in the final multivariable model. It can also be hypothesized that squirrels have different foraging behaviour in deciduous forest than in other habitats, which may influence their risk of exposure to *T. gondii*. Other parameters, including most categories of land use, the presence of domestic cats, and the availability of food, were not found to be significantly associated with *T. gondii* infection. This may have be due to the number of samples examined in this study, which was relatively low for modelling purposes. Also, cat density data were only available at the regional level, which may not have always reflected cat density at the locations where the squirrels were found. Data on human density and distance to urban area were available at a higher resolution and might therefore have been better proxies for cat density.

As information on toxoplasmosis in red squirrels is limited, it is impossible to know whether the situation determined in the present study is comparable to those of neighbouring countries with similar habitats and climatic conditions. In comparison to available data for the rest of Europe, the infection rates of *T. gondii* determined for Eurasian red squirrels in the present study, and especially for 2014 (52.9%), were relatively high. For instance, infection rates were notably lower in squirrels in Jersey [2.1% (7/337)] [75] and Finland [16% (3/19)] [18]. The discrepancies between these results may indicate that the high proportion of infected squirrels observed in 2014 was an exception. In sum, we are unable to provide a comprehensive explanation for the sudden upsurge in the number of *T. gondii*-related deaths in 2014 and its subsequent decline based on the predictor variables determined in the present study.

## Conclusions

*Toxoplasma gondii* type II was observed in 27.5% of the Eurasian red squirrels examined. Most of the infected squirrels had pneumonia and hepatitis. MS typing revealed *T. gondii* type II genotypes similar to those previously reported for Europe. Factors associated with oocyst survival rather than squirrel feeding behaviour were found to be putative risk factors of infection, which suggests that the death of squirrels due to infection with *T. gondii* is likely indicative of a high environmental load of infectious oocysts. The findings of this study contribute to our understanding of the epidemiology of *T. gondii*, and indicate the importance of this parasite as a cause of death in Eurasian red squirrels in the Netherlands.

### Supplementary Information

**Additional file 1: Table S1. **Sequences of primers, probes, and final concentrations used in the quantitative real-time qPCR. **Table S2.** Description, spatial resolution and source of explanatory variables included in the analysis to assess risk factors for *Toxoplasma gondii* quantitative real-time qPCR positivity in squirrels. **Table S3.** Geographical origin of *Toxoplasma gondii* quantitative real-time qPCR-positive Eurasian red squirrels; proportion per region and per province. **Table S4**. *Hammondia hammondi* quantitative real-time qPCR-positive samples per year, month, and province. **Table S5**. Grouped and detailed causes of death implicated by pathological examination compared to quantitative real-time qPCR results for *Toxoplasma gondii*.** Table S6. **Results of IHC and quantitative real-time qPCR for *Toxoplasma gondii* and *Hammondia hammondi. ***Table S7. **Samples positive for *Toxoplasma gondii* by IHC versus those positive by IHC but negative by quantitative real-time qPCR, per year. **Table S8. **Results of the univariable mixed logistic regression used to assess risk factors for *Toxoplasma gondii* quantitative real-time qPCR positivity in squirrels (*n* = 175 squirrels).** Table S9. **Assessment of multicollinearity for variables included in the final model, as determined by variance inflation factor.

**Additional file 2: Figure S1. **Living Planet Index (LPI) per region (north sandy region, central sandy region, south sandy region, and other regions of the Netherlands). **Figure S2**. Correlation matrix between predictor variables selected by univariable analysis to assess risk factors for *Toxoplasma gondii* quantitative real-time qPCR positivity in squirrels. **Figure S3.** Results of a *Toxoplasma gondii*-specific quantitative real-time qPCR for lung, liver, and heart samples. The Wilcoxon rank sum test was restricted to data on squirrels in which *T. gondii* qPCR was positive for all of the three organs (*n* = 35). Cq values of heart samples were significantly higher than those of the liver and lung, and those of the lung were significantly higher than those of the liver (Wilcoxon rank sum test, *P* < 0.001). **Figure S4.** Fig. S4A: Number of *Toxoplasma gondii*- and *Hammondia hammondi*-positive samples per month. Fig. S4B: Proportion of *Toxoplasma gondii*- and *Hammondia hammondi*-positive samples per month. Samples were grouped into the following categories: *T. gondii* positive (*Tgo*), *H. hammondi* positive (*Hha*), *T. gondii* and *H. hammondi* positive (*Tgo&Hha*), negative in qPCR (*Neg*). **Figure S5.** Fig. S5A: Number of *Toxoplasma gondii*- and *Hammondia hammondi*-positive samples per year. Fig. S5B: Proportion of *Toxoplasma gondii-* and *Hammondia hammondi*-positive samples per year. Samples were grouped into the following categories: Tgo, Hha, Tgo&Hha, Neg. **Figure S6. **Statistically significant correlation (*P* < 0.001) of Cq values obtained for different organs of squirrels by *Toxoplasma gondii* quantitative real-time qPCR, i.e. liver vs. lung [residual SE (RSE), 2.432, *df* 33, multiple* R*^2^ 0.5753, adjusted* R*^2^ 0.5624,* F* 44.69, *P* < 0.001; Fig. S6A], heart vs. lung (RSE, 1.477,* df* 33, multiple* R*^2^ 0.7469, adjusted* R*^2^ 0.7392,* F* 97.36, *P* < 0.001; Fig. S6B), and heart vs. liver (RSE, 2.109,* df* 33, multiple* R*^2^ 0.4837, adjusted* R*^2^ 0.468,* F* 30.91, *P* < 0.001; Fig. S6C). Adjusted* R*^2^ values were obtained by linear regression (regression line and 95% CIs are displayed).

**Additional file 3: Dataset S1.** Population genetic analyses of *Toxoplasma gondii* MS typing data.

**Additional file 4: Dataset S2.** Squirrels in the Netherlands.

## Data Availability

The data used in this study are available from the Additional files.

## Abbreviations

APC: Affinity propagation clustering
BBG: Functional land use map
CI: Confidence interval
COD: Cause of death
DWHC: Dutch Wildlife Health Centre
EGFP: Enhanced green fluorescent protein
FD: Freezing days, minimum temperature ≤ − 6 ℃
GLMM: Generalized linear mixed model
IHC: Immunohistochemical analyses
IQR: Interquartile range
LPI: Living Planet Index
MLG: Multi-locus genotype
MS: Microsatellite
MSN: Minimum spanning network
qPCR: Quantitative real-time PCR
R25mm: Heavy rainfall days, mean precipitation ≥ 25 mm
SU25: Summer days, maximum temperature > 25 ℃
