# Supplementary material for: Drivers of infection with Toxoplasma gondii genotype type II in Eurasian red squirrels (Sciurus vulgaris)
Source: Parasit Vectors. 2024 Jan 23;17:30. doi: 10.1186/s13071-023-06068-6 (PMC10804655; doi:10.1186/s13071-023-06068-6)
Supplement: Supplementary file 1 — Additional file 1: Table S1. Sequences of primers, probes, and final concentrations used in the quantitative real-time qPCR. Table S2. Description, spatial resolution and source of explanatory variables included in the analysis to assess risk factors for Toxoplasma gondii quantitative real-time qPCR positivity in squirrels. Table S3. Geographical origin of Toxoplasma gondii quantitative real-time qPCR-positive Eurasian red squirrels; proportion per region and per province. Table S4. Hammondia hammondi quantitative real-time qPCR-positive samples per year, month, and province. Table S5. Grouped and detailed causes of death implicated by pathological examination compared to quantitative real-time qPCR results for Toxoplasma gondii. Table S6. Results of IHC and quantitative real-time qPCR for Toxoplasma gondii and Hammondia hammondi. Table S7. Samples positive for Toxoplasma gondii by IHC versus those positive by IHC but negative by quantitative real-time qPCR, per year. Table S8. Results of the univariable mixed logistic regression used to assess risk factors for Toxoplasma gondii quantitative real-time qPCR positivity in squirrels (n = 175 squirrels). Table S9. Assessment of multicollinearity for variables included in the final model, as determined by variance inflation factor. [file 13071_2023_6068_MOESM1_ESM.docx]

**Additional file 1**

**Additional file 1: Table S1.** Sequences of primers, probes, and final concentrations used in real-time PCR.

| Targeted species or DNA | Primer/Probe | Sequence 5’-3’ | Calculated melting temperature (Tm) | Final concentration | Template volume | Cycling conditions | Reference |
| --- | --- | --- | --- | --- | --- | --- | --- |
| *Toxoplasma gondii* | ToxoTal-F | TGG TTG GGA AGC GAC GAG AG | 61.4°C | 0.8 µM | 1 µl | 50°C, 2 min; 95°C, 10 min; 45 x (95°C, 15 sec; 60°C, 1 min) | [1] |
|  | ToxoTal-R | CAT CAC GAG GAA AGC GTC | 56.0°C | 0.8 µM |  |  |  |
|  | ToxoTal-Probe [LNA] | FAM-AG[+A] GA[+C] AC[+C] GG[+A] ATG CG[+A] T-BHQ1 |  | 0.2 µM |  |  |  |
| *Hammondia hammondi* | Hham275F | CTA CAA GGG GAG CGT CCT CG | 63.4°C | 0.5 µM | 1 µl | 95°C, 5 min; 45 x (95°C, 10 sec; 60°C, 30 sec) | [2] |
|  | Hham81R | GAG GAG AGT CGG AGA GGG AG | 63.4°C | 0.5 µM |  |  |  |
|  | Hham222P | Cy5-GTG GAA AGA CTG AAG CCG GA-BHQ2 | 59.4°C | 0.16 µM |  |  |  |
| Internal control targeting a EGFP gene-based plasmid | EGFP1-F | GAC CAC TAC CAG CAG AAC AC | 59.4°C | 0.15 µM |  | Conditions of PCRs targeting *T. gondii* or *H. hammondi* | [3] |
|  | EGFP2-R | GAA CTC CAG CAG GAC CAT G | 58.8°C | 0.15 µM |  |  |  |
|  | EGFP1-Probe | HEX-AGC ACC CAG TCC GCC CTG AGC A-BHQ1 | 67.7°C | 0.1 µM |  |  |  |

**Additional file 1: Table S2.** Description, spatial resolution, and source of explanatory variables included in the analysis to assess risk factors for *Toxoplasma gondii* real-time PCR positivity in squirrels.

| Variable | Definition | Spatial resolution of original data source | Data source |
| --- | --- | --- | --- |
| Sex | Sex of the squirrel (e.g., female, male) |  | DWHC |
| Domestic cat population | Number of domestic cats adjusted per region (i.e., North, East, South, West). |  | Dibevo and Dutch Food Industry Companion Animals (NVG); [4] |
| Human population density | Human population density within a 3 km diameter buffer. | 500 m | Statistics Netherlands (CBS) |
| Nearest distance to urban areas | Nearest Euclidean to distance to urban areas in meters. | 1 ha | Statistics Netherlands (CBS) |
| Farm density | Number of farms within 3 km diameter buffer. |  | Basic Registration Commercial Register (NHR), Chamber of Commerce (KVK) |
| Mast year | Mast year score of oak and beech per year. |  | [5] |
| Fungi year | Living planet index score per habitat type per year based on saprotrophic and ectomycorrhiza species (n = 88). |  | Network Ecological Monitoring (NEM); Statistics Netherlands (CBS); Dutch Mycological Association (NMV) |
| Land-use | Proportion (%) of agricultural, urban, deciduous forest, coniferous forest, infrastructure, nature reserves, and water areas within a 3 km diameter buffer. | 25 m^2^ | Wageningen Environmental Research, Wageningen University and Research (WUR). |
| Nearest distance to water | Nearest Euclidean to distance to water in meters. | 1 ha | Statistics Netherlands (CBS) |
| CDD | Maximum number of consecutive dry days (precipitation < 1 mm) preceding the date of specimen collection (time lag interval of 3, 6, and 12 months) [6]. | 1 km^2^ | Royal Netherlands Meteorological Institute (KNMI) |
| SU25 | Number of days when maximum temperature >25 ⁰C preceding the date of specimen collection (period of 2 weeks, 1, 3, 6, and 12 months) [6]. | 1 km^2^ | Royal Netherlands Meteorological Institute (KNMI) |
| R25mm | Number of days when the daily precipitation > 25 mm preceding the date of specimen collection (period of 2 weeks, 1, 3, 6, and 12 months) [6]. | 1 km^2^ | Royal Netherlands Meteorological Institute (KNMI) |
| FD6 | Number of days when minimum temperature ≤ -6 ⁰C preceding the date of specimen collection (period of 2 weeks, 1, 3, 6, and 12 months) [6]. | 1 km^2^ | Royal Netherlands Meteorological Institute (KNMI) |

**Additional file 1: Table S3**. Geographical origin of *Toxoplasma gondii* real-time PCR-positive Eurasian red squirrels; proportion per region and per province.

| Region in the Netherlands | Province | *T. gondii* positives/ number examined | Proportion of positives (%) | 95% CI |
| --- | --- | --- | --- | --- |
| East | Gelderland | 12/52 | 23.1 | 12.5 – 36.8 |
|  | Overijssel | 11/25 | 44.0 | 24.4 – 65.1 |
| North | Drenthe | 8/26 | 30.8 | 14.3 – 51.8 |
|  | Fryslân | 0/1 | 0.0 | 0.0 – 97.5 |
|  | Groningen | 2/5 | 40.0 | 5.2 – 85.3 |
| South | Limburg | 1/7 | 14.3 | 0.3 – 57.9 |
|  | North Brabant | 10/32 | 31.2 | 16.1 – 50.0 |
| West | Flevoland | 0/1 | 0.0 | 0.0 – 97.5 |
|  | North Holland | 2/11 | 18.2 | 2.3 – 51.8 |
|  | Utrecht | 3/16 | 18.8 | 4.0 – 45.6 |
|  | South Holland | 0/2 | 0.0 | 0.0 – 84.2 |

**Additional file 1: Table S4**. *Hammondia hammondi* real-time PCR positive samples per year, month, and province.

| Year | Month | Province | % Positive samples (positive/total) |
| --- | --- | --- | --- |
| 2014 | September | Overijssel | 2.9% (1/34) |
| 2015 | November | Gelderland | 3.7% (1/27) |
| 2016 | May | Groningen | 5.0% (1/20) |
| 2017 | June | Drenthe | 6.7% (1/15) |
| 2020 | March | North Brabant | 3.0% (1/33) |

**Additional file 1: Table S5**. Grouped and detailed causes of death implicated in pathological examination relative to real-time PCR results for *Toxoplasma gondii***.**

| Grouped COD - Fig. 2 | Detailed COD | Positive  *T. gondii* | Negative  *T. gondii* |
| --- | --- | --- | --- |
| Hepatitis | Hepatitis | 1 | 0 |
| Inflammation multiple organs | Hepatitis, pneumonia | 7 | 2 |
| Inflammation multiple organs | Hepatitis, pneumonia, splenitis | 3 | 0 |
| Inflammation multiple organs | Hepatitis, myocarditis, splenitis | 2 | 0 |
| Inflammation multiple organs | Hepatitis, pneumonia, splenitis, myocarditis | 2 | 0 |
| Inflammation multiple organs | Enteritis, peritonitis | 1 | 0 |
| Inflammation multiple organs | Hepatitis, pneumonia, encephalitis | 1 | 0 |
| Inflammation multiple organs | Hepatitis, pneumonia, lymphadenitis | 1 | 0 |
| Inflammation multiple organs | Hepatitis, pneumonia, myocarditis | 1 | 0 |
| Inflammation multiple organs | Hepatitis, pneumonia, myocarditis, encephalitis | 1 | 0 |
| Inflammation multiple organs | Hepatitis, thymus necrose | 1 | 0 |
| Inflammation multiple organs | Liver necrosis, pneumonia, myocarditis | 1 | 0 |
| Inflammation multiple organs | Pneumonia, hepatitis, splenitis | 1 | 0 |
| Inflammation multiple organs | Pneumonia, myocarditis, encephalitis, dermatitis | 1 | 0 |
| Inflammation multiple organs | Pneumonia, splenitis, encephalitis | 1 | 0 |
| Inflammation multiple organs | Hepatitis, pneumonia, pleuritis, lymphadenitis | 0 | 1 |
| Inflammation multiple organs | Pneumonia, myocarditis | 0 | 1 |
| Inflammation multiple organs | Pneumonia, splenitis, trauma | 0 | 1 |
| Pneumonia | Pneumonia | 6 | 12 |
| Sepsis ^a^ | Sepsis | 1 | 5 |
| Trauma | Trauma | 11 | 66 |
| Other | Other | 0 | 7 |
| Unclear | Unclear | 6 | 34 |

**^a^** Sepsis was in most cases the result of infection with *Pasteurella multocida* (4/6)*.*

**Additional file 1:** **Table S6.** Results of IHC and real-time PCR for *Toxoplasma gondii* and *Hammondia hammondi.*

|  |  | Real-time PCR positive | Real-time PCR negative |
| --- | --- | --- | --- |
| *T. gondii* | IHC positive for *T. gondii* | 15 | 28 |
|  | IHC negative for *T. gondii* | 1 | 12 |
|  | IHC not tested for *T. gondii* | 33 | 89 |
| *H. hammondi* | IHC positive for *T. gondii* | 1 | 42 |
|  | IHC negative for *T. gondii* | 0 | 13 |
|  | IHC not tested for *T. gondii* | 4 | 118 |

**Additional file 1: Table S7.** Samples positive for *Toxoplasma gondii* by IHC versus those positive by IHC but negative by real-time PCR, per year.

| Year | IHC | Positive - PCR | Negative - PCR |
| --- | --- | --- | --- |
| 2014 | Positive | 4 (100%) | 0 (0%) |
| 2015 | Positive | 3 (60%) | 2 (40%) |
| 2016 | Positive | 2 (50%) | 2 (50%) |
| 2017 | Positive | 2 (28.6%) | 5 (71.4%) |
| 2018 | Positive | 1 (33.3%) | 2 (66.7%) |
| 2019 | Positive | 2 (25%) | 6 (75%) |
| 2020 | Positive | 1 (8.3%) | 11 (91.7%) |

**Additional file 1: Table S8.** Results from the univariable mixed logistic regression used to assess risk factors for *Toxoplasma gondii* real-time PCR positivity in squirrels (*n* = 175 squirrels).

|  | Univariable regression | |  |
| --- | --- | --- | --- |
| Variables ^a^ | ***P*-value** | **OR (95% CI)** | |
| Cat population | 0.300 | 0.80 (0.52 – 1.22) | |
| Human population density | **0.153** | **1.28 (0.91 – 1.79)** | |
| Nearest distance to urban areas | **0.055** | **0.58 (0.34 – 1.01)** | |
| Farm density | 0.312 | 0.82 (0.55 – 1.21) | |
| Land-use – % Agricultural | 0.720 | 0.94 (0.66 – 1.33) | |
| Land-use – % Urban | **0.151** | **1.29 (0.91 – 1.81)** | |
| Land-use – % Deciduous Forest | **0.101** | **0.72 (0.49 – 1.07)** | |
| Land-use – % Coniferous Forest | 0.579 | 0.90 (0.63 – 1.30) | |
| Land-use – % Infrastructure | **0.193** | **1.26 (0.89 – 1.77)** | |
| Land-use – % Nature reserve | 0.772 | 0.95 (0.67 – 1.34) | |
| Land-use – % Water | 0.302 | 1.19 (0.86 – 1.65) | |
| Nearest distance to water bodies | **0.031** | **0.60 (0.38 – 0.96)** | |
| Nearest distance to park | 0.207 | 0.79 (0.54 – 1.14) | |
| CDD – 2 weeks (2-week period preceding sampling date) | **0.170** | **1.27 (0.90 – 1.78)** | |
| CDD – 1 month (1-month period preceding sampling date) | 0.572 | 1.11 (0.77 – 1.62) | |
| CDD – 3 months (3-month period preceding sampling date) | 0.569 | 1.12 (0.76 – 1.64) | |
| CDD – 6 months (6-month period preceding sampling date) | 0.754 | 0.93 (0.60 – 1.44) | |
| CDD – 12 months (12-month period preceding sampling date) | 0.568 | 0.87 (0.55 – 1.39) | |
| R25mm – 2 weeks (2-week period preceding sampling date)^¥^ | - | - | |
| R25mm – 1 month (1-month period preceding sampling date) | 0.886 | 1.03 (0.73 – 1.44) | |
| R25mm – 3 months (3-month period preceding sampling date) | 0.937 | 0.99 (0.69 – 1.40) | |
| R25mm – 6 months (6-month period preceding sampling date) | 0.736 | 1.06 (0.74 – 1.52) | |
| R25mm – 12 months (12-month period preceding sampling date) | **0.031** | **1.48 (1.04 – 2.11)** | |
| SU25 – 2 weeks (2-week period preceding sampling date) | **0.007** | **0.31 (0.13 – 0.72)** | |
| SU25 – 1 month (1-month period preceding sampling date) | **0.020** | **0.51 (0.29 – 0.90)** | |
| SU25 – 3 months (3-month period preceding sampling date) | 0.791 | 1.05 (0.72 – 1.55) | |
| SU25 – 6 months (6-month period preceding sampling date) | 0.466 | 1.15 (0.78 – 1.70) | |
| SU25 – 12 months (12-month period preceding sampling date) | 0.345 | 0.80 (0.50 – 1.27) | |
| FD6 – 2 weeks (2-week period preceding sampling date) | 0.790 | 0.95 (0.64 – 1.40) | |
| FD6 – 1 month (1-month period preceding sampling date) | 0.849 | 0.96 (0.66 – 1.40) | |
| FD6 – 3 months (3-month period preceding sampling date) | 0.869 | 1.03 (0.72 – 1.48) | |
| FD6 – 6 months (6-month period preceding sampling date) | **0.047** | **0.56 (0.32 – 0.99)** | |
| FD6 – 12 months (12-month period preceding sampling date) | **0.236** | **0.74 (0.45 – 1.22)** | |
| Mast year – Oak | **0.073** | **0.65 (0.41 – 1.04)** | |
| Mast year – Beech | 0.906 | 1.03 (0.60 – 1.76) | |
| Fungi year | 0.465 | 1.21 (0.73 – 2.00) | |
| Fungi year x Mast year – Oak ^b^ | – | – | |
| Fungi year x Mast year – Beech | 0.790 | 0.93 (0.52 – 1.64) | |

## Model random effects, σ^2^ (random effect variance): 3.29; Marginal R^2^ / Conditional R^2^:

^¥^ Univariable logistic regression for the variable R25mm – 2 weeks was not feasible due to zero variance.

^a^ For details on variables, refer to Additional file 1: Table S2

^b^ Univariate logistic regression with the interaction term between fungi year and mast year (oak) was not possible due to a boundary (singular) fit issue.

**Additional file 1: Table S9.** Assessment of multicollinearity for variables included in the final model, as determined by Variance inflation factor (VIF).

| Variables | VIF | 95% CI lower limit | 95% CI upper limit |
| --- | --- | --- | --- |
| Land-use – % Deciduous Forest | 1.05 | 1.00 | 1.98 |
| FD6 – 6 months (6 months period preceding sampling date) | 1.14 | 1.04 | 1.50 |
| R25mm – 12 months (12 months period preceding sampling date) | 1.09 | 1.01 | 1.56 |
| SU25 – 2 weeks (2 weeks period preceding sampling date) | 1.05 | 1.00 | 1.92 |
| Mast year – Oak | 1.19 | 1.07 | 1.52 |
| Nearest distance to urban areas | 1.05 | 1.00 | 2.02 |
| CDD – 2 weeks (2 weeks period preceding sampling date) | 1.15 | 1.04 | 1.50 |

**References**

1. Talabani, H., et al., *Contributions of immunoblotting, real-time PCR, and the Goldmann-Witmer coefficient to diagnosis of atypical toxoplasmic retinochoroiditis.* Journal of clinical microbiology, 2009. **47**(7): p. 2131-2135.

2. Schares, G., et al., *A real-time quantitative polymerase chain reaction for the specific detection of Hammondia hammondi and its differentiation from Toxoplasma gondii.* Parasites & Vectors, 2021. **14**(1): p. 1-11.

3. Hoffmann, B., K. Depner, H. Schirrmeier, and M. Beer, *A universal heterologous internal control system for duplex real-time RT-PCR assays used in a detection system for pestiviruses.* Journal of virological methods, 2006. **136**(1-2): p. 200-209.

4. Borst, N., et al., *Feiten & Cijfers Gezelschapsdierensector. 2011.* HAS Kennistransfer, Hogeschool HAS Den Bosch, 2011. **186568**.

5. Swart, A., et al., *Modelling human Puumala hantavirus infection in relation to bank vole abundance and masting intensity in the Netherlands.* Infection Ecology & Epidemiology, 2017. **7**(1): p. 1287986.

6. ETCCDI. *Climate Change Indices*. 2020 [cited 2023 22-02-2023]; Available from: <http://etccdi.pacificclimate.org/list_27_indices.shtml>.
