# Supplementary material for: Drivers of infection with Toxoplasma gondii genotype type II in Eurasian red squirrels (Sciurus vulgaris)
Source: Parasit Vectors. 2024 Jan 23;17:30. doi: 10.1186/s13071-023-06068-6 (PMC10804655; doi:10.1186/s13071-023-06068-6)
Supplement: Supplementary file 2 — Additional file 2: Figure S1. Living Planet Index (LPI) per region (north sandy region, central sandy region, south sandy region, and other regions of the Netherlands). Figure S2. Correlation matrix between predictor variables selected by univariable analysis to assess risk factors for Toxoplasma gondii quantitative real-time qPCR positivity in squirrels. Figure S3. Results of a Toxoplasma gondii-specific quantitative real-time qPCR for lung, liver, and heart samples. The Wilcoxon rank sum test was restricted to data on squirrels in which T. gondii qPCR was positive for all of the three organs (n = 35). Cq values of heart samples were significantly higher than those of the liver and lung, and those of the lung were significantly higher than those of the liver (Wilcoxon rank sum test, P < 0.001). Figure S4. Fig. S4A: Number of Toxoplasma gondii- and Hammondia hammondi-positive samples per month. Fig. S4B: Proportion of Toxoplasma gondii- and Hammondia hammondi-positive samples per month. Samples were grouped into the following categories: T. gondii positive (Tgo), H. hammondi positive (Hha), T. gondii and H. hammondi positive (Tgo&Hha), negative in qPCR (Neg). Figure S5. Fig. S5A: Number of Toxoplasma gondii- and Hammondia hammondi-positive samples per year. Fig. S5B: Proportion of Toxoplasma gondii- and Hammondia hammondi-positive samples per year. Samples were grouped into the following categories: Tgo, Hha, Tgo&Hha, Neg. Figure S6. Statistically significant correlation (P < 0.001) of Cq values obtained for different organs of squirrels by Toxoplasma gondii quantitative real-time qPCR, i.e. liver vs. lung [residual SE (RSE), 2.432, df 33, multiple R2 0.5753, adjusted R2 0.5624, F 44.69, P < 0.001; Fig. S6A], heart vs. lung (RSE, 1.477, df 33, multiple R2 0.7469, adjusted R2 0.7392, F 97.36, P < 0.001; Fig. S6B), and heart vs. liver (RSE, 2.109, df 33, multiple R2 0.4837, adjusted R2 0.468, F 30.91, P < 0.001; Fig. S6C). Adjusted R2 values were obtained by linear re [file 13071_2023_6068_MOESM2_ESM.docx]

**Additional file 2**

**
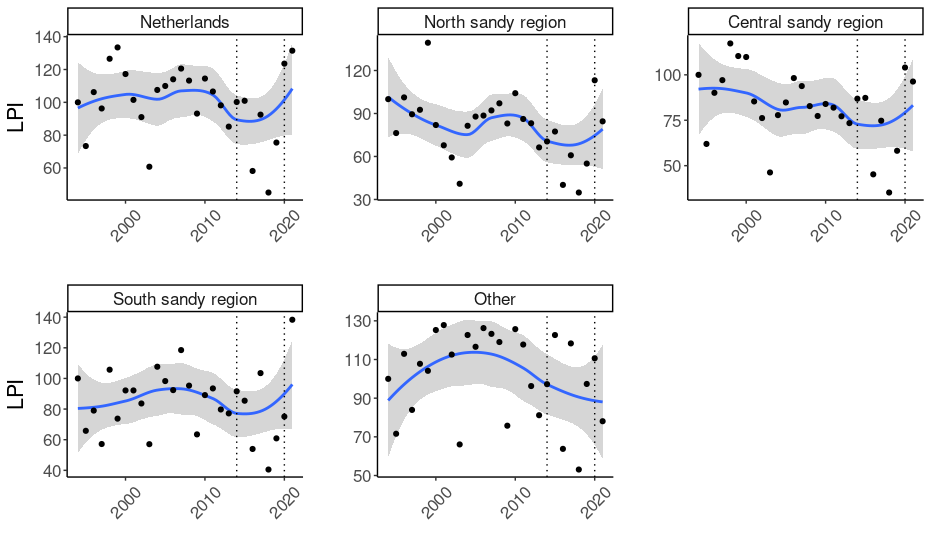
**

**Additional file 2: Fig. S1.** Living planet index (LPI) per region (north sandy region, central sandy region, south sandy region, and other regions of the Netherlands).


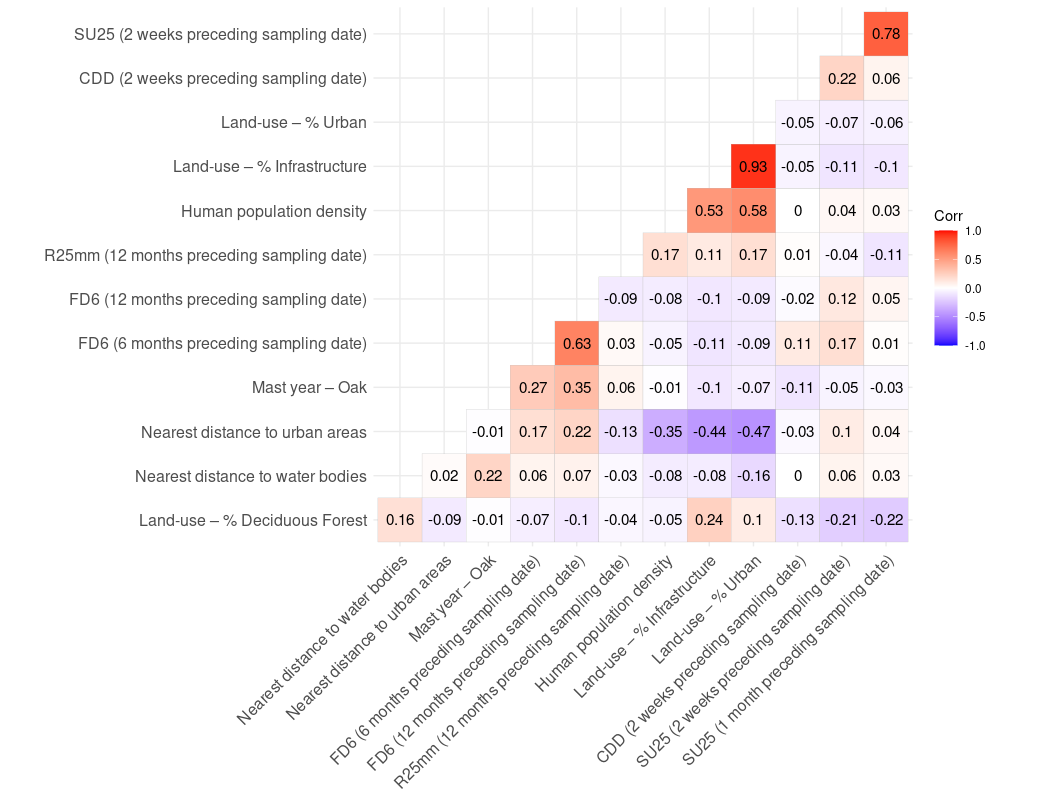


**Additional file 2: Fig. S2**. Correlation matrix between predictor variables selected by univariable analysis to assess risk factors for *Toxoplasma gondii* real-time PCR positivity in squirrels.


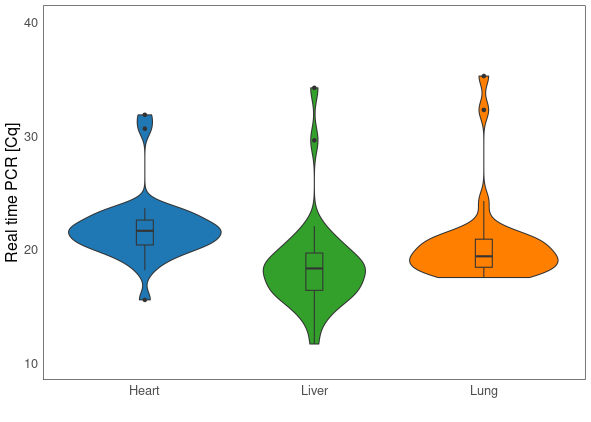


**Additional file 2: Fig. S3.** Results in a *Toxoplasma gondii* specific real-time PCR in lung, liver, and heart samples. The Wilcoxon rank sum test was restricted to data on squirrels in which *T. gondii* PCR was positive for all the three organs (n=35). Cq values in heart samples were significantly higher than those of liver and lung, and those of the lung were significantly higher than those of the liver (Wilcoxon rank sum test, *P* < 0.001).


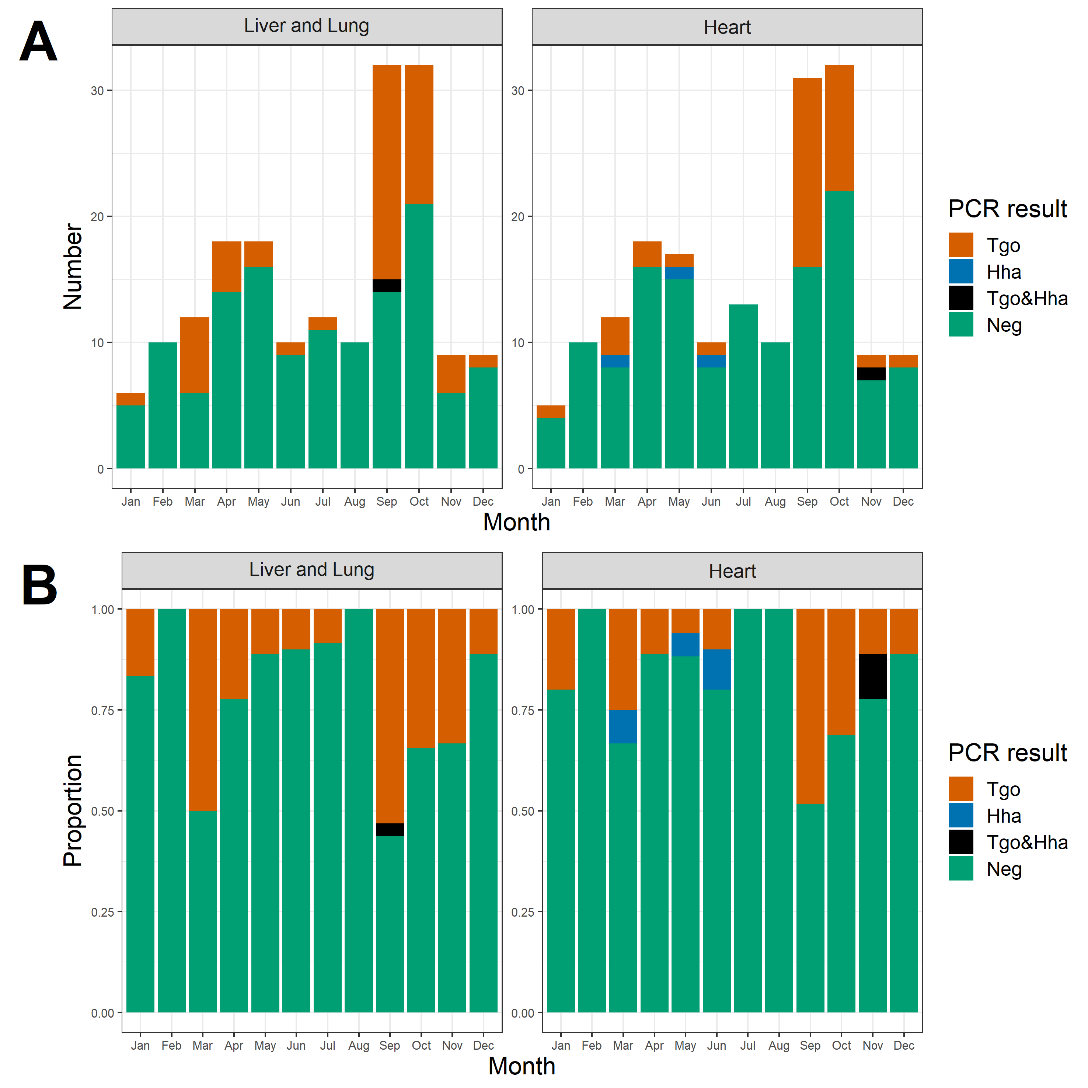


**Additional file 2: Fig. S4.** **Fig. S4A**: Number of *Toxoplasma gondii*, and *Hammondia hammondi* positive samples per month. **Fig. S4B**: Proportion of *T. gondii-*, and *H. hammondi*-positive samples per month. Samples were grouped into the following categories: *T. gondii* positive (*Tgo*); *H. hammondi* positive (*Hha*); *T. gondii* and *H. hammondi* positive (*Tgo&Hha*); negative in PCR (*Neg*).


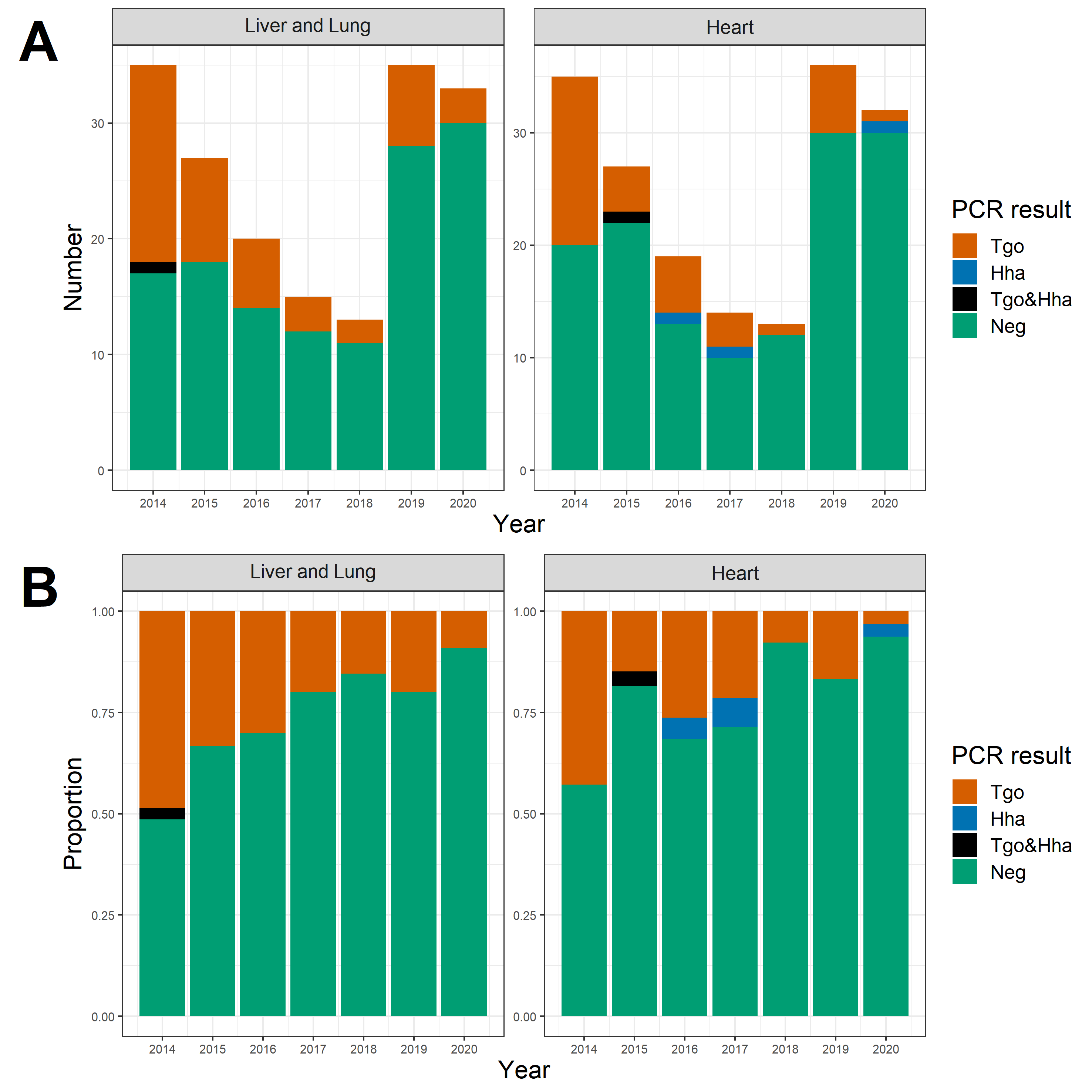


**Additional file 2: Fig. S5.** **Fig. S5A**: Number of *Toxoplasma gondii*, and *Hammondia hammondi* positive samples per year. **Fig. S5B**: Proportion of *T. gondii-*, and *H. hammondi*-positive samples per year. Samples were grouped into the following categories: Tgo, Hha, Tgo&Hha, Neg.


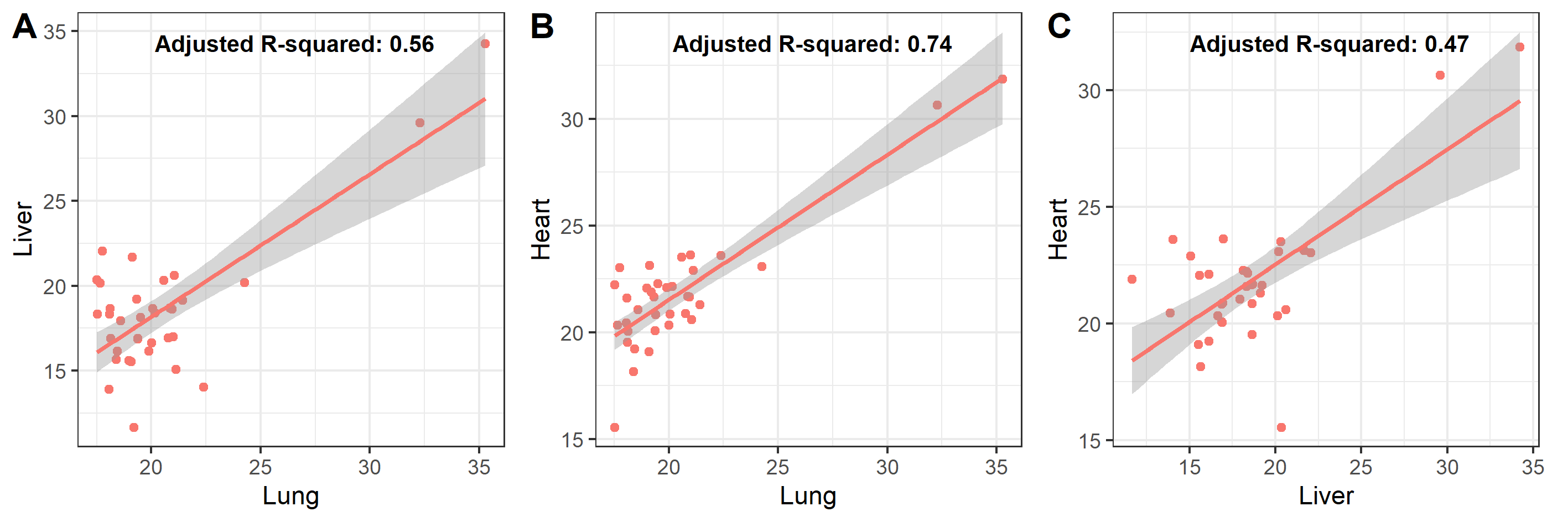
 **Additional file 2: Fig. S6.** Statistically significant correlation (*P* < 0.001) of Cq values obtained in squirrels for different organs by the *Toxoplasma gondii* real-time PCR, i.e., liver vs. lung [residual SE (RSE), 2.432, *df* 33, multiple *R^2^* 0.5753, adjusted *R^2^*: 0.5624, *F* 44.69, *P*-value < 0.001; **Fig. S6A**], heart vs. lung (RSE, 1.477, *df* 33, multiple *R^2^* 0.7469, adjusted *R^2^*: 0.7392, *F* 97.36, *P*-value < 0.001; **Fig. S6B**), and heart vs. liver (RSE, 2.109, *df* 33, multiple *R^2^* 0.4837, adjusted *R^2^* 0.468, *F* 30.91, *P*-value < 0.001; **Fig. S6C**). Adjusted *R^2^* values were obtained by linear regression (i.e., regression line and 95% confidence intervals are displayed).
